# Grafts and Patches: Optimized but Not Optimal Materials for Congenital Heart Surgery

**DOI:** 10.1007/s00246-023-03153-6

**Published:** 2023-04-10

**Authors:** Armin Darius Peivandi, Sven Martens, Boulos Asfour, Sabrina Martens

**Affiliations:** 1 Department of Cardiothoracic Surgery, University Hospital Muenster, Muenster, Germany; 2 Department of Pediatric Cardiac Surgery, University Hospital Bonn, Bonn, Germany; 3 Department of Cardiothoracic Surgery, Division of Pediatric Cardiac Surgery, University Hospital Muenster, Muenster, Germany

**Keywords:** Congenital heart surgery, Patch, Conduit

## Abstract

A variety of materials are available for the surgery of children with congenital heart defects. In addition to growth-related mismatch, degeneration of the material in particular frequently leads to reoperation. Therefore, the choice of conduits and patches should be made carefully. This article provides an overview of the most commonly implanted materials in pediatric cardiac surgery.

Structural changes can be detected in all available materials. Depending on the age at implantation and the site of implantation, the extent and time course of material degeneration vary. Autologous material is still the gold standard in reconstructive surgery. Biological materials have largely replaced artificial materials in clinical use.

The search for the ideal material continues. In pediatric cardiac surgery, there are only optimized but no optimal materials.

## Introduction

Complex congenital heart defects usually require surgical treatment in infancy or early childhood. In many cases, either human (autologous, homologous), animal (bovine, porcine, equine), or artificial (DACRON®, DuPont Corporation, Wilmington, USA or GORE-TEX®, W.L. Gore & Associates, Inc., Newark, Delaware, USA) materials are used for reconstructive procedures in pediatric cardiac surgery. These materials exhibit varying durability depending on the implantation site and surgical technique. For example, patches (pericardial patches) degenerate differently than conduits (conduits with and without valves, such as Goretex). Degeneration of a variety of these materials results in frequent reoperations in childhood.

This review focuses on most commonly used materials that are mainly commercially available. The authors are fully aware of tissue-engineered center-specific modified patches, conduits, and valves [1]. Due to the heterogeneity of these materials, however, they cannot be covered in depth as part of this review.

## Clinical Background

The prevalence of congenital heart defects is approximately 1% of live births. While ventricular and atrial septal defects are generally the most common congenital heart malformations (VSD (48.9%), ASD (17%)), the PAN study identified tetralogy of Fallot (TOF) (2.5%) and transposition of the great arteries (2.2%) as the most common complex cyanotic heart defects [2]. In most of these cardiac defects, correction is performed without planned reoperation. In some children, however, reoperations are required as part of multilevel therapy or because of growth-related mismatch, and in others, foreign material must be replaced, due to calcifications, pseudo-aneurysm formation, neo-atherosclerosis like lesions, and degeneration of collagen and elastic fibers [3, 4]. Children with congenital heart defects usually receive pediatric cardiological care and regular follow-up; as adults, they are referred to specialized outpatient clinics. In addition to regular physical examinations, echocardiography is a central component of imaging diagnostics. Degenerative changes can also be assessed by MRI and CT [5].

In the following, the problem of frequent reoperations in childhood mainly due to material degeneration is exemplified by the treatment options of TOF patients:

TOF describes the presence of the following four components: pulmonary stenosis, right ventricular hypertrophy, ventricular septal defect, and overriding aorta [6].

Today, surgical TOF correction is usually performed within the first year of life [6, 7]. The operation includes closure of the VSD and reconstruction of the right ventricular outflow tract (RVOT) [8]. Depending on the anatomic circumstances of the individual case (pulmonary stenosis, pulmonary atresia, absent pulmonary valve, size of the pulmonary valve annulus, coronary artery anatomy), surgical management of the RVOT may vary between infundibular myectomy, commissurotomy, patch expansion (transannular patch), and complete reconstruction with a conduit [6–9].

In most cases, valve-sparing surgery or transannular patchplasty are the preferred surgical options. However, reconstructive surgery with conduits is sometimes unavoidable.

Homografts (human prostheses from a donor bank) or Contegra® grafts (bovine jugular veins) are used for RVOT reconstruction. Degeneration of such valved prostheses due to calcification or fibrous deposits leads to stenosis and insufficiency, which may ultimately lead to early reoperation [3]. However, degenerative developments are not the only cause for graft replacement. In this context, somatic outgrowths leading to functional stenosis should also be mentioned.

No matter the cause, frequent reoperations in childhood result in an increased risk of surgery and a negative impact on the quality of life (physical, psychological, and overall). Withdrawal behavior, attention deficits, and externalization problems have been identified as psychological consequences [10].

## Overview of Commonly Used Materials in Pediatric Cardiac Surgery

Table 1 provides an overview of some of the most commonly used materials in congenital heart surgery.

**Table 1:** Conduits, heart valves, and patches commonly used in pediatric cardiac surgery (random order, without claiming completeness)

Conduits	
Cardiac homografts	Tissue Banks (e.g., European Homograft Bank)
Contegra® graft	Medtronic plc, Dublin, Ireland
Hancock® conduit	Medtronic plc, Dublin, Ireland
Freestyle™ prosthesis	Medtronic plc, Dublin, Ireland
Biopulmonic conduit	Biointegral Surgical, Inc., Mississauga, ON
Heart valves	
Prosthetic heart valves	• Open Pivot by Medtronic • SJM Regent™ by Abbott • On-X by CryoLife
Biological heart valves	• Perimount Magna Ease by Carpentier-Edwards • SJM Trifecta™ by Abbott
Patches	
Autologous pericardium	–
Bovine pericardial patches	• CardioCel patch by LeMaitre Vascular • Peri-Guard® by LaMed
Porcine pericardial patch	Curved NoReact Patch by BioIntegral
Porcine submucosa patch	CorPatch® by CorMatrix® Cardiovascular
Equine pericardial patch	Matrix Patch™ by Autotissue
Artificial patches	GORE-TEX® by Gore Medical DACRON® by DuPont Corporation

### Conduits

Cardiac *homografts* are human grafts obtained from the left or right ventricular outflow tract of donors. Homografts are usually processed in specialized tissue banks where they are available on demand. While there are a number of officially accredited tissue banks in Europe (e.g., Hannover, Berlin, Bad Oeynhausen, Barcelona), many centers like ours rely on the European Homograft Bank in Brussels.

In 1989, the European Homograft Bank was founded as an internationally cooperating and non-profit organization [11]. Aortic and/or pulmonary explants are systematically processed there [12]. After standardized morphological assessment, professional preparation, and triple antibiotic treatment (vancomycin, lincomycin, polymyxin B), cryopreservation is performed [13].

The grafts are available to cardiac surgeons from all over Europe and can be ordered as needed (elective surgery or emergency surgery).

Due to the declining donor numbers and the resulting lack of available tissue in small sizes (10% of requests do not receive a positive vote) [14], alternative prostheses are increasingly gaining access to the medical device market. In this context, the *Contegra®* prosthesis occupies an exposed position.

The Contegra® graft (Medtronic plc, Dublin, Ireland), available since the 1990s, is a bovine jugular vein containing a tri-leaflet valve and processed in glutaraldehyde. In contrast to the homograft, the industrially produced Contegra® is often available in small sizes (diameter 12–22 mm, length 12–15 cm) [15].

In addition to the conduits described in detail above, further alternatives commercially available on the market must be mentioned. These include the *Hancock®* conduit (porcine aortic valve sewn into a woven conduit) [16, 17], the *Freestyle* prosthesis (porcine aortic root) (both (Medtronic plc, Dublin, Ireland)) [18, 19], or the *BioPulmonalConduit* (BioIntegral Surgical, Mississauga, Canada) [20].

### Prosthetic and Biological Heart Valves

If possible, heart valve replacement is avoided in younger children. The first surgical attempt is usually rather reconstructive surgery of the valves. However, in cases where valve replacement is necessary (failed reconstruction attempts, teenagers), valve replacement is mainly performed using mechanical valves. Nowadays, these valves are double-wing heart valve prostheses. Many manufacturers have launched their products in the past. Common examples include but are not limited to Medtronic Open Pivot [21], St. Jude Medical Regent™ [22] and On-X. The last mentioned prosthesis was introduced especially for pediatric use as this valve requires lower Warfarin levels [23].

All mechanical valve prostheses carry bleeding risks as they require life-long anticoagulation. On the contrary, biological prostheses are almost exclusively used in pulmonary position due to their limited durability and to avoid thrombosis under low-pressure physiology. In the RVOT, e.g., Carpentier-Edwards Perimount Magna Ease [24, 25] or St. Jude Medical Trifecta™ prostheses [26, 27] can be used.

### Patches

Pericardial patches currently used in pediatric cardiac surgery are described below:

*Autologous* pericardium is obtained from the same patient during surgery and implanted (if necessary, a brief treatment with 0.625% glutaraldehyde for 20 min and subsequent careful irrigation is performed) [28]. It currently represents the gold standard for reconstructive surgery, e.g., reconstruction of aortic valve leaflets [29].

The use of human material is limited, especially in re-do surgery when autologous pericardium cannot be used any more. In former days, fixation with glutaraldehyde was routinely performed, but studies now suggest a negative impact on tissue durability [30]. Non-glutaraldehyde-fixed pericardium is less pliable for surgical use. As a result, other biological materials have become popular. They mimic human pericardium but do not resemble the exact structure of their human counterparts.

The most common alternative to autologous pericardium is *bovine* pericardium. This material is produced by various manufacturers. The company LeMaitre Vascular (Sulzbach, Germany) offers the CardioCel patch, which has undergone a special anticalcification process (removal of cell particles and nucleic acids and minimization of glutaraldehyde content) [31]. According to the manufacturer, a special tear resistance characterizes other bovine patches from LaMed (Oberhaching, Germany). They are described as acellular [32].

*Equine* patches are also available (Matrix Patch™, Autotissue, Berlin, Germany). According to the manufacturer, they are made of decellularized horse pericardium (patches with low DNA content) [33].

Also of animal origin is the BioIntegral Curved NoReact Patch (BioIntegral Surgical, Mississauga, Canada), a curved *porcine* pericardial patch [34].

While pericardium is the most commonly used biomaterial for patch manufacturing, it is not the only one: the CorPatch® (CorMatrix® Cardiovascular, Roswell, Georgia, USA) is made from *porcine small intestinal submucosa*. In a porcine ischemic heart disease model, the material improved functional recovery of the myocardium [35]. In addition to the use of this material for epicardial infarct repair in adult patients, its performance has also been studied in children with congenital heart defects: No ingrowth of native cardiac tissue was detected after 21 months when the patch was used as a hemi-Fontan baffle [36].

*Artificial* materials have increasingly taken a back seat in pediatric reconstructions due to the multitude of biological alternatives. In earlier days, DACRON or GORE-TEX® were the materials that were mainly used. As thromboembolic complications along with short-term infections became evident in DACRON patches [37], this material was gradually replaced. Furthermore, tissue processing technology had improved and made biological materials more attractive to use. GORE-TEX®, a polytetrafluoroethylene patch that is also known in the textile industry for its durability, is used almost exclusively as a pericardial patch in chest closure for adhesion prophylaxis.

## Degeneration as a Cause of Reoperations–Clinic and Histology

**Fig. 1:**
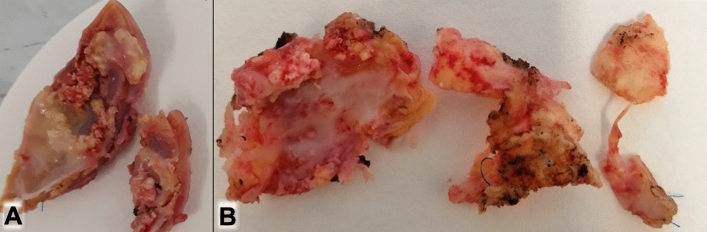
Strongest, already macroscopically visible, calcifications in explants: **A**: Contegra® graft; **B**: homograft

**Fig. 2:**
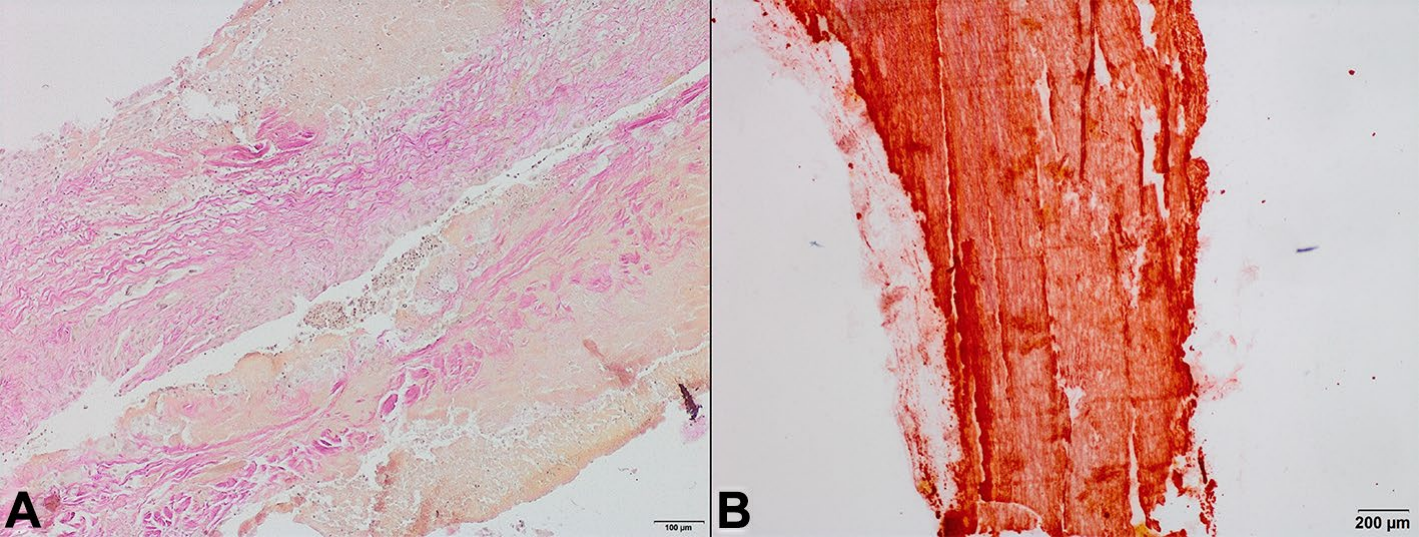
**A**: Inflammation in the context of endocarditis of a Contegra.® graft (EvG staining). **B**: Selective visualization of calcification of a homograft (Alizarin red staining)

Degenerative changes are observed in all materials. They may occur at different time points after implantation and vary in their severity. When becoming apparent, they lead to reoperations. Macroscopic findings of explants from reoperations are shown in Fig. 1. Figure 2 shows inflammation and calcification as the most common cause of implant failure, in addition to growth-related mismatch, which inevitably leads to reoperations, too.

Implantation of homografts is still the strategy of choice in patients requiring reconstruction, e.g., of the RVOT [38]. *Pulmonary* homografts show very good mid-term results: the rate of reoperation in the first 8 years after implantation was only 7.8% in a recently published study (mean age of patients 26.1 ± 13.6 years) [39]. However, pulmonary homografts can present with clinical insufficiencies and obstruction, which may lead to graft replacement within 4–6 years in some patients [15]. *Aortic* homografts in pulmonary position are also prone to fibro-calcification, especially in patients younger than 3 years. This may be due to the lower elastin content of aortic homografts [40, 41].

Fiore et al. published their surgical results in infants and young children < 2 years of age and reported that 59% of their patients required reoperation due to high-grade stenosis and/or pulmonary insufficiencies [41]. Thus, the youngest patients are particularly affected by the problem of reoperation. Histologically, homograft failure is caused by intimal hyperplasia on the one hand [42], and calcifications, ossifications, and chronic immune reactions in the adventitia on the other hand [43, 44].

As a good surgical alternative to homografts, the Contegra® prosthesis has been generally accepted in the last 3 decades. This was mainly due to its easy surgical handling and availability in small sizes. A European multicenter study showed good 7-year results with respect to explant rates, endocarditis, stenosis, insufficiencies, and other events [45]. Compared to the homograft, the prosthesis shows a comparable hemodynamic performance. Its natural morphology has a positive influence on the surgical outcome [15, 46].

**Fig. 3:**
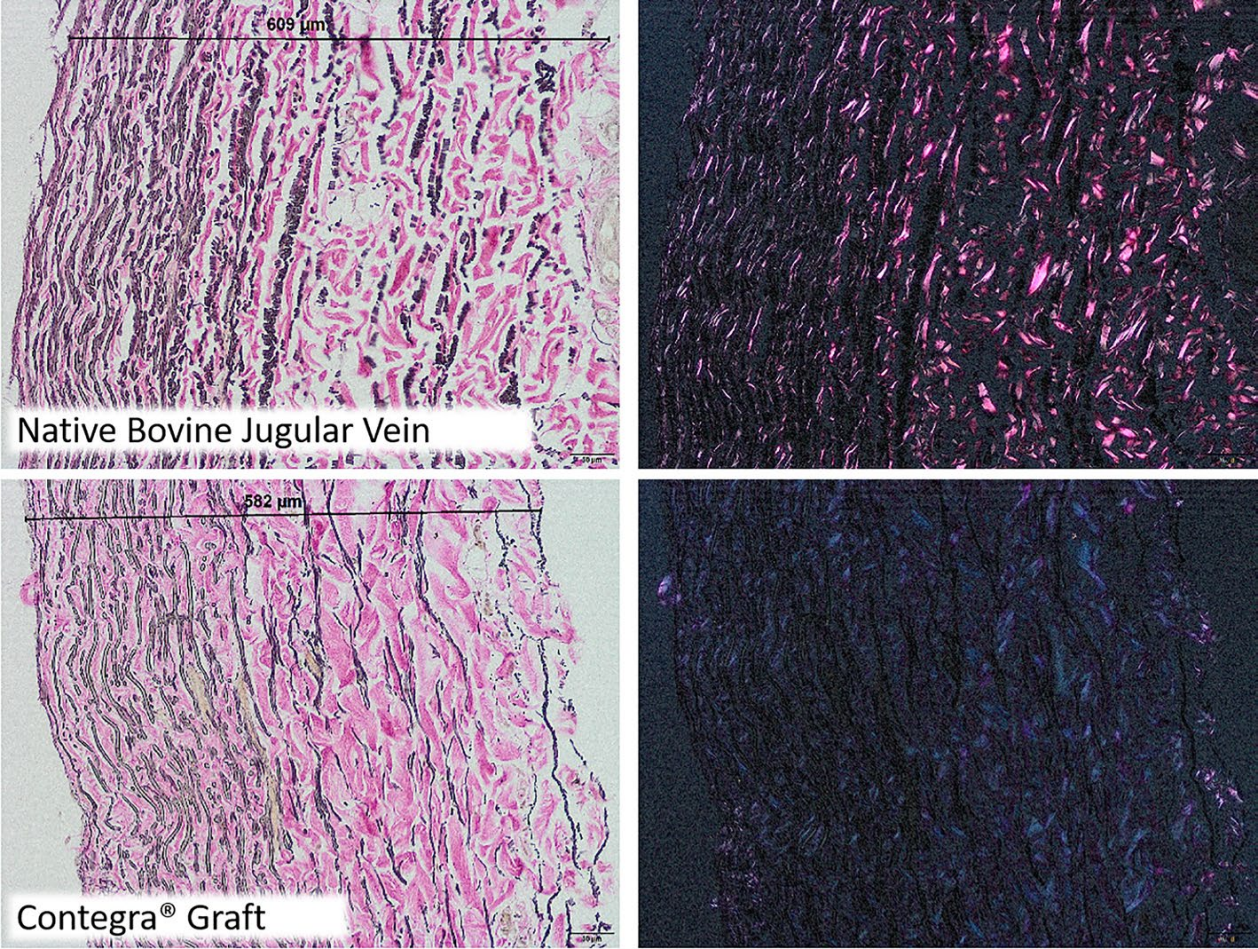
Degeneration (loosened tissue, reduced number of elastic fibers and partially destroyed collagen network) caused by the production process: top: native jugular vein of a young bull with intact birefringence in polarization image (right image) and bottom: Contegra.® graft with significantly reduced birefringence (right image: polarization)

Nevertheless, there are also critical voices regarding the use of Contegra® grafts: Doubled reoperation rates compared to homografts are described [47], as well as infections of the prosthesis and problems with high RV-LV pressures and with small sizes [48, 49]. In the latter, stenotic fibrotic membranes and neointimal proliferation at the distal anastomosis are repeatedly observed [4, 50, 51]. The formal pathomechanism of Contegra® degeneration is based on elastic degeneration with accompanying stiffness of the prosthesis as well as on intimal hyperplasia with neointimal calcifications and heterotopic ossifications [3]. The polarization microscopic image of Contegra® reveals structural alterations caused by its production process (Fig. 3).

Patches represent a more heterogeneous graft group because they are derived from different species. Furthermore, their surgical use is more diverse.

Autologous pericardium can be harvested from any patient who has not undergone a large number of previous operations before. For many years, glutaraldehyde treatment of the harvested pericardium brought the advantages of better surgical handling due to higher mechanical stability, at the expense of an increased tendency to calcification [52]. Currently, the once established fixation method has therefore been abandoned in many clinics.

Severe calcification is the main cause of clinical degeneration of bovine pericardium used as a transannular patch in Fallot correction. Pseudo-aneurysmal changes and consequent pulmonary valve insufficiency are also observed.

Severe calcification is the main cause of clinical degeneration of bovine pericardium, which is often used as a transannular patch in TOF correction. Pseudo-aneurysmal changes and consequent pulmonary valve regurgitation are also observed. However, it is not just the degenerative change of the patch itself that necessitates reoperation, but the clinical increase in pulmonary insufficiency and consecutive right heart strain.

When used in reconstructive surgery of the aortic valve, echocardiographically immobile, thickened tissue portions can be seen after a longer period of time. This may lead to stenosis [53]. Histological correlates are calcifications and connective tissue deposits [54].

Currently, there are no reliable randomized clinical trials on equine pericardium. The manufacturer’s website refers to case reports [33]. The use of equine pericardium is justified by animal studies in which neither negative structural changes nor calcifications could be detected [55]. A recently published retrospective cohort study showed first good short-term clinical results [56], although no systematic distinctions were made between implantation site and congenital heart defect.

Porcine patches complete the animal product line and show promising clinical results [57]. A large-scale histological evaluation is also pending.

## Conclusions and Future Challenges

Degeneration can be observed clinically in both conduits and patches used in pediatric cardiac surgery. It often results in unavoidable reoperations. However, not only the material as such, but also the implantation site and the surgical technique, as well as the age of the child seem to have an influence on long-term outcome.

To date, the ideal material for use in pediatric cardiac surgery has not been identified [58] as only optimized but no optimal materials are available.

Large-scale prospective randomized studies and systematic histopathological workups are desirable. New emerging techniques such as 3D printing, computational modeling, and tissue engineering may help providing individualized treatment options with optimal geometrical and flow properties in future [59].

## Data Availability

The data underlying this article will be shared on reasonable request to the corresponding author.
